# Supplementary material for: Risk of Liver Cirrhosis and Hepatocellular Carcinoma after Fontan Operation: A Need for Surveillance
Source: Cancers (Basel). 2020 Jul 6;12(7):1805. doi: 10.3390/cancers12071805 (PMC7408507; doi:10.3390/cancers12071805)
Supplement: Supplementary file 1 [file cancers-12-01805-s001.pdf]

cancers

MDPI

Article

Risk of Liver Cirrhosis and Hepatocellular Carcinoma after Fontan Operation: A Need for Surveillance

Jun Sik Yoon [1,2,†], Dong Ho Lee [3,†], Eun Ju Cho [1,*,‡], Mi Kyoung Song [4], Young Hun Choi [3], Gi Beom Kim [4], Yun Bin Lee [1], Jeong-Hoon Lee [1], Su Jong Yu [1], Haeryoung Kim [5], Yoon Jun Kim [1], Jung-Hwan Yoon [1] and Eun Jung Bae [4,*,‡]

1 Department of Internal Medicine and Liver Research Institute, Seoul National University College of Medicine, Seoul 03080, Korea; yojusi@naver.com (J.S.Y.); yunbin@hanmail.net (Y.B.L.); pindra@empal.com (J.-H.L.); ydoctor2@hanmail.net (S.J.Y.); yoonjun@snu.ac.kr (Y.J.K.); yoonjh@snu.ac.kr (J.-H.Y.)
2 Department of Internal Medicine, Busan Paik Hospital, Inje University College of Medicine, Busan 47392, Korea
3 Department of Radiology, Seoul National University College of Medicine, Seoul 03080, Korea; dhlee.rad@gmail.com (D.H.L.); choiyounghun@gmail.com (Y.H.C.)
4 Division of pediatric cardiology, Department of Pediatrics, Seoul National University Children's Hospital, Seoul National University College of Medicine, Seoul 03080, Korea; mksong52@hanmail.net (M.K.S.); ped9526@snu.ac.kr (G.B.K.)
5 Department of Pathology, Seoul National University College of Medicine, Seoul 03080, Korea; medannabel@gmail.com
* Correspondence: creatio3@snu.ac.kr (E.J.C.); eunjbaek@snu.ac.kr (E.J.B.); Tel.: +82-2-2072-2242 (E.J.C.); +82-2-2072-3097 (E.J.B.); Fax: +82-2-762-9662 (E.J.C.); +82-2-743-3455 (E.J.B.)
† These two authors contributed equally to this work.
‡ These two authors are corresponding authors.

Supplementary

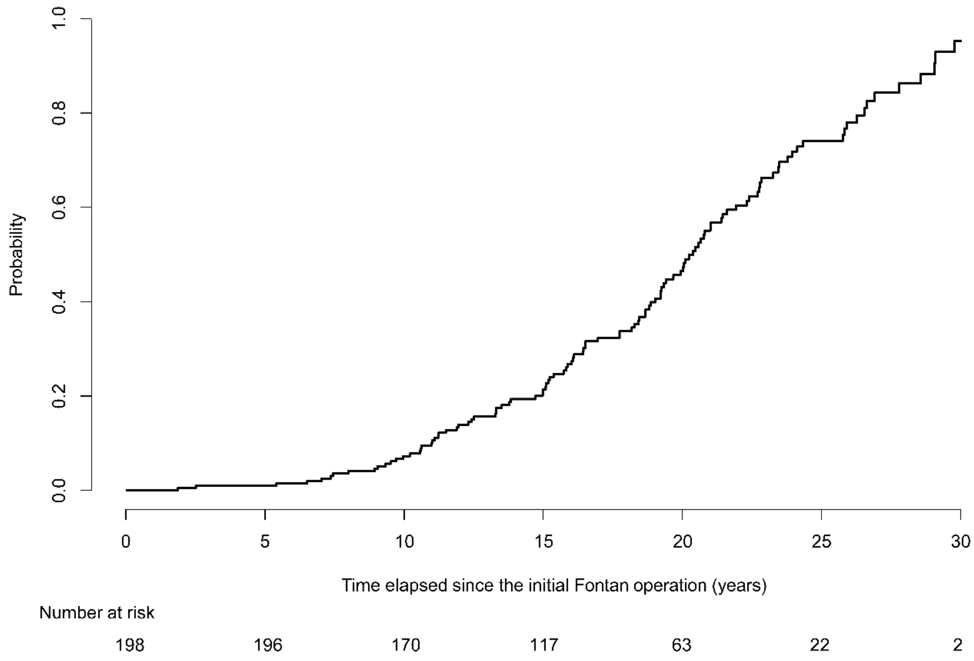

Figure S1. The cumulative incidence rate of cirrhosis in a subgroup of patients who were followed up within 3 years interval after the Fontan operation.

The cumulative incidence rates of cirrhosis at 5, 10, 20, and 30 years after the Fontan operation were 1%, 7.3%, 46.5, and 95.3%, respectively. The cumulative incidence rate increased gradually until 10 years after the Fontan operation and then increased rapidly thereafter.

Table S1. Comparisons of demographics and clinical characteristics between patients diagnosed with and without cirrhosis after the Fontan operation.

	**Patients Diagnosed with Cirrhosis (*n* = 221)**	**Patients Diagnosed without Cirrhosis (*n* = 92)**	***p* Value**
Male gender	146 (66.1%)	58 (63.0%)	0.70
Age at Fontan operation (years)	2.8 (2.1-4.1)	2.6 (2.1-3.6)	0.13
Type of Fontan operation			<0.001
Atriopulmonary connection	49 (22.2%)	5 (5.4%)	
Lateral tunnel	95 (43.0%)	31 (33.7%)	
Extracardiac	77 (34.8%)	56 (60.9%)	
Time elapsed since the initial Fontan operation (years)	16.9 (12.6-21.0)	15.1 (12.1-20.4)	0.13
Development of HCC	7 (3.2%)	0 (0.0%)	0.19
Post-operative arrhythmia	67 (30.3%)	20 (21.7%)	0.16
Protein losing enteropathy	22 (10.0%)	0 (0.0%)	0.004
HBV infection	4 (1.9%)	1 (1.1%)	1
HCV infection	2 (0.9%)	0 (0.0%)	0.88
Platelet count ($x10^3/\mu L$)	157.0 (115.0-204.0)	201.0 (178.0-248.0)	<0.001
AST (U/L)	26.0 (21.0-31.0)	24.0 (20.0-27.0)	0.004
ALT (U/L)	23.0 (16.0-30.0)	21.0 (18.0-28.0)	0.66
GGT (U/L)	62.0 (42.5-90.5)	51.5 (33.0-75.0)	0.03
Cholesterol (mg/dL)	139.0 (120.0-153.0)	138.5 (120.5-151.0)	0.87
Spleen diameter (cm)	11.4 (10.2-12.8)	10.1 (9.0-10.9)	<0.001
MELD-XI	8.0 (4.8-11.1)	6.0 (3.3-8.9)	<0.001
Forn's index	4.4 (3.2-5.8)	3.4 (1.9-4.3)	<0.001
APRI	0.4 (0.3-0.6)	0.3 (0.2-0.4)	<0.001
FIB-4	0.2 (0.1-0.3)	0.1 (0.1-0.1)	<0.001

Data are expressed as median (interquartile range), or n (%). * The data were collected at the time of diagnosis of cirrhosis for patients with cirrhosis and at the time of last imaging study for patients without cirrhosis. Abbreviations: AST, aspartate aminotransferase; ALT, alanine aminotransferase; GGT, gamma-glutamyltransferase; MELD-XI, Model for End-stage Liver Disease excluding INR; APRI, aspartate transaminase to platelet ratio index; FIB-4, Fibrosis-4 index.

cancers

MDPI

Table S2. Comparisons of demographics and clinical characteristics measured at the time of cardiac catheterization between patients diagnosed with and without cirrhosis after the Fontan operation.

	**Patients diagnosed with cirrhosis (*n* = 53)**	**Patients diagnosed without cirrhosis (*n* = 79)**	**Total (n = 132)**	***p* Value**
Age at the initial Fontan operation (years)	2.8 (2.0-5.6)	2.4 (1.8-3.4)	2.6 (2.0-3.7)	0.051
Male sex	39 (73.6%)	51 (64.6%)	90 (68.2%)	0.368
Type of Fontan operation				0.23
Atriopulmonary connection	15 (28.3%)	13 (16.5%)	28 (21.2%)	
Lateral tunnel	21 (39.6%)	40 (50.6%)	61 (46.2%)	
Extracardiac	17 (32.1%)	26 (32.9%)	43 (32.6%)	
Time elapsed since the initial Fontan operation (years)	18.6 (13.3-23.3)	10.6 (7.1-16.2)	13.7 (8.7-19.7)	<0.001
Fontan pressure (mmHg)	13.0 (11.0-16.0)	13.0 (11.0-15.0)	13.0 (11.0-15.0)	0.704
VEDP (mmHg)	9.0 (6.0-13.0)	8.0 (6.0-10.0)	9.0 (6.0-12.0)	0.11
SaO_2 (%)	91.8 (87.0-94.0)	93.0 (89.7-95.0)	92.4 (88.9-94.9)	0.027
Warfarin	26 (49.1%)	26 (32.9%)	52 (39.4%)	0.093
Diuretics	23 (43.4%)	24 (30.4%)	47 (35.6%)	0.178
ACEI/ARB	39 (73.6%)	60 (75.9%)	99 (75.0%)	0.918
Post-operative arrhythmia	23 (43.4%)	19 (24.1%)	42 (31.8%)	0.032
Protein losing enteropathy	4 (7.5%)	10 (12.7%)	14 (10.6%)	0.518
Viral hepatitis	1 (1.9%)	4 (5.1%)	5 (3.8%)	0.637
Platelet count ($x10^3/\mu L$)	164.0 (123.0-208.0)	209.0 (151.5-253.0)	191.0 (141.0-245.0)	0.002
Total bilirubin (mg/dL)	1.5 (1.0-2.4)	0.9 (0.6-1.6)	1.1 (0.7-1.7)	<0.001
AST (U/L)	26.0 (23.0-30.0)	29.0 (23.0-38.0)	27.0 (23.0-33.0)	0.053
ALT (U/L)	22.0 (16.0-27.0)	19.0 (15.0-25.0)	20.0 (16.0-26.0)	0.336
GGT (U/L)	72.0 (53.0-102.0)	51.0 (32.0-82.0)	60.0 (40.0-90.5)	0.003
Albumin (g/dL)	4.5 (4.2-4.7)	4.4 (4.1-4.6)	4.4 (4.2-4.6)	0.069
MELD-XI	9.2 (4.9-11.5)	5.6 (0.5-8.8)	6.8 (2.7-10.4)	<0.001
Forn's index	4.7 (2.8-6.2)	2.0 (-0.1-3.7)	3.0 (1.0-5.2)	<0.001
APRI	0.4 (0.3-0.6)	0.4 (0.3-0.5)	0.4 (0.3-0.5)	0.115
FIB-4	0.2 (0.1-0.3)	0.1 (0.1-0.2)	0.1 (0.1-0.2)	<0.001

Data are expressed as median (interquartile range), or n (%). Abbreviations: VEDP, ventricular end-diastolic pressure; SaO_2, arterial oxygen saturation; ACEI, angiotensin-converting enzyme inhibitor; ARB, angiotensin II receptor blocker; AST, aspartate aminotransferase; ALT, alanine aminotransferase; GGT, gamma-glutamyltransferase; MELD-XI, Model for End-stage Liver Disease excluding INR; APRI, aspartate transaminase to platelet ratio index; FIB-4, Fibrosis-4 index.

Table S3. Characteristics of 7 patients with HCC after the Fontan operation.

**A. Baseline characteristics**						
**Case no.**	**Sex**	**Age at HCC diagnosis (years)**	**Time elapsed from the Fontan operation to HCC diagnosis (years)**	**Type of the Fontan operation**	**Modality of HCC diagnosis**	Arrhythmia
1	Female	37.7	28.9	APC	Clinical features	Yes
2	Male	21.2	21.7	LT	Clinical features	No
3	Male	19.5	16.5	APC	Histology	Yes
4	Male	36.2	30.9	APC	Histology	Yes
5	Female	35.3	27.6	APC	Histology	Yes
6	Male	27.5	21.1	APC	Clinical features	Yes
7	Female	17.0	8.2	LT	Histology	Yes
B. Radiologic finding						
Case no.	Number of tumor	Largest tumor diameter (cm)	Washout	Surface nodularity	Size of spleen (cm)	Presence of collateral vessels
1	Single	1.0	DP only	Yes	10.6	Yes
2	Single	2.1	PVP and DP	Yes	11.3	Yes
3	Multiple	2.5	DP only	Yes	16.6	Yes
4	Single	4.0	PVP and DP	Yes	10.7	Yes
5	Single	4.1	PVP and DP	Yes	10.1	Yes
6	Single	4.8	PVP and DP	Yes	14.0	Yes
7	Multiple	10.5	PVP and DP	Yes	12.8	Yes
C. Laboratory finding and treatment modality						
Case no.	Platelet ($x10^3/\mu L$)	AFP before treatment (ng/mL)	AFP after the first treatment (ng/mL)	Treatment modality	Survival time (months)	Status
1	238000	39.6	9.7	TACE	42.4	Survival
2	122000	160.6	4.1	TACE	17.6	Survival
3	55000	6.0	5.7	TACE	163.4	Death
4	145000	247.4	27.9	Resection	0.8	Survival
5	81000	720.0	14.3	TACE	31.1	Survival
6	107000	141.6	6.0	TACE	126.0	Survival
7	186000	211580	743500.0	Supportive	49.6	Death

Abbreviations: HCC, hepatocellular carcinoma; APC, atriopulmonary connection; EC, extracardiac; LT, lateral tunnel; ALT, alanine aminotransferase; AFP, alpha-fetoprotein; TACE, transarterial chemoembolization.

cancers MDPI

Table S4. Diagnostic performance of different washout criteria on CT in 30 patients with arterial hyperenhancement nodule larger than 1 cm.

Imaging criteria	Sensitivity	Specificity	PPV	NPV
Arterial phase Hyperenhancement				
+ washout on PVP only	71.4% (5/7)	100% (23/23)	100% (5/5)	92.0% (23/25)
+ washout on PVP and/or DP	100% (7/7)	52.2% (12/23)	28.9% (7/18)	100% (12/12)

Abbreviations: PPV, positive predictive value; NPV, negative predictive value; PVP, portal venous phase; DP, delayed phase.
